# Investigation of muscle-specific beef color stability at different ultimate pHs

**DOI:** 10.5713/ajas.19.0943

**Published:** 2020-02-25

**Authors:** Shuang Wu, Jina Han, Rongrong Liang, Pengcheng Dong, Lixian Zhu, David L. Hopkins, Yimin Zhang, Xin Luo

**Affiliations:** 1 Lab of Beef Processing and Quality Control, College of Food Science and Engineering, Shandong Agricultural University, Taian, Shandong 271018, China; 2 NSW Department of Primary Industries, Centre for Red Meat and Sheep Development, PO Box 129, Cowra NSW 2794, Australia

**Keywords:** Beef Color, Dark Cutting, Oxygen Consumption, Metmyoglobin Reducing Activity, Ultimate pH

## Abstract

**Objective:**

This study was aimed to investigate the muscle-specific beef color stability at normal and high ultimate pHs.

**Methods:**

The impact of muscle (*Longissimus lumborum* [LL] vs *psoas major* [PM]) and pH (normal ultimate pH [Np] vs high pH dark cutting beef [Hp]) on color stability, indicated by basic color traits, metmyoglobin reducing activity (MRA) and oxygen consumption (OC), as well as the lipid oxidation, were determined over 7 days of display at 4°C.

**Results:**

Hp-LL had the highest pH (6.92), followed by Hp-PM (6.01), Np-PM (5.76), and Np-LL (5.52). Hp-LL had increased (p<0.05) a*, chroma and % oxymyoglobin during display. Hp-LL also had the highest metmyoglobin (MMb) reducing activity and OC among all the samples, thus, the greatest color stability, although very dark throughout storage, with lowest values for lightness (L*) and yellowness (b*). Np-LL also exhibited relatively high color stability, as a result of its lower % MMb and OC and higher MRA than *psoas* muscle samples. The 0.2 unit difference of the pH between Hp and Np psoas muscle, resulted in the difference of the color intensity, not the color stability. Interestingly, high pH *psoas* muscle (Hp-PM) did not have better color stability than Np-PM, and in fact had lower color stability than even Np-LL. The similar level of OC and lipid oxidation cannot explain the difference in color stability between Hp-PM and Np-LL.

**Conclusion:**

The Hp does not always show better color stability compared with Np beef, which depends on the muscle type. The balance of MRA and OC is important to keep the color in great intensity and stability in the meantime.

## INTRODUCTION

Meat color is one of the most important quality traits influencing consumer purchasing decisions, since consumers generally evaluate freshness and wholesomeness of meat through surface color [1]. Fresh beef color is mainly determined by myoglobin content and its redox state. Deoxymyoglobin (DMb) exists as a purplish-red color usually occurring on fresh cut surfaces, and it converts to a bright cherry red when exposed to air or oxygen (oxymyoglobin [OMb]). However, metmyoglobin (MMb) can lead to a brown color through the oxidation of ferrous myoglobin [2]. Generally, dark cutting beef is rejected by most consumers due to appearance. In fact, brownish discoloration or dark color caused a loss of $1 billion and $165 to $170 million, respectively, to the US beef industry in 2000 [3,4].

Of several endogenous factors influencing beef color and color stability, muscle type and ultimate pH have received significant attention. Several previous studies have reported that meat color stability is a muscle-specific trait, due to the variable proportion of metabolic fiber types (oxidative or glycolytic) among muscles [5,6]. Compared with color-stable *Longissimus lumborum* (LL), biochemical studies have documented that *psoas major* (PM) is a color-labile muscle [7,8]. Dark cutting beef is associated with a high ultimate pH (Hp), and the increased ultimate pH enhances water-holding capacity and mitochondrial activity, which lead to less light scattering and the formation of more DMb than normal pH (Np) beef, resulting in a darker surface color [9–11]. Interestingly, although Hp has less redness intensity, it demonstrates greater oxygen consumption (OC), and notably greater color stability than Np beef [12,13].

Several research groups have investigated the color values of different muscles from Hp beef. Some study reported that dark cutting status had various effects on color L*, a*, b* values of hindquarter muscles at 7 days postmortem [14]. Holman and Hopkins [15] compared the eating quality traits of aged bolar blade, striploin and topside cuts of Hp beef, and found that the largest dark cutting effect was in the striploin. However, they did not compare the interaction effects of cut (muscle type) and ultimate pH on color traits and color stability, and especially, the underlying mechanisms of those color differences.

Therefore, in this study, the color stability comparison of LL and PM from Np and Hp beef, under aerobic packaging during retail display was studied. This was to ascertain the order of color stability between different muscles, which will provide more information on the underlying mechanisms of color stability as affected by muscle type and pHu.

## MATERIALS AND METHODS

### Animal and sample preparation

Beef carcasses (24 months old, Luxi Yellow×Simmental cattle, 300 to 350 kg carcass weight) were randomly selected from a commercial abattoir. Fourteen carcasses were assigned to 2 groups based on observed muscle pH at approximately 24 h postmortem: Hp beef (also called dark, firm and dry beef, which exhibits a significantly darker color and higher pH value compared with normal beef. In China, the threshold of Hp beef was defined as 6.1 [16]) group (pH>6.1, n = 7) and Np value group (pH 5.4 to 5.8, n = 7). Muscle pH was collected on the anterior surface of LL between the 12–13th rib on the right side of each carcass using a portable pH meter (Senven2Go-S2, Mettler-Toledo, Greifensee, Switzerland), which was calibrated with two buffers (pH 4.0 and 7.0); each carcass was measured twice. LL and PM were removed from right carcass sides at 48 h postmortem (chilling at 0°C to 4°C), commercially vacuum packaged, and then transported to the laboratory on ice.

Following overnight storage, LL and PM from the same carcass were fabricated into ten 2.5-cm-thick steaks, and eight steaks were randomly assigned to day 0, 3, 5, and 7 for retail display (two steaks on each day as duplicates). The remaining 2 steaks were randomly assigned to day 0 and 7 for measuring total viable microbial count. Steaks were placed on foam trays with absorbent pads (DLS-25, Sealed Air Corp., Danbury, CT, USA), overwrapped with Polyethylene (PE) film (water vapor permeability: 23.5 g/m^2^/24 h, oxygen transmission rate: 16,654 cm^3^/m^2^/24 h/atm), and stored in a walk-in cooler at 2°C±1°C under continuous lighting (1,600 to 2,000 lx, Leishi Warm Yellow Light-Emitting Diode Light; color temperature = 3,000 K). All steaks were rotated daily to minimize the variance in light intensity or temperature caused by the location. The following traits were measured at each time point: pH, surface color traits (L*, a*, b*, Chroma and hue values, R630/580, relative myoglobin content), OC, and metmyoglobin reducing activity (MRA). Samples for subsequent lipid oxidation analysis were obtained at each sampling time-point and stored at −80°C.

### Proximate components and pH

Moisture, protein and fat content of the steaks was determined based on National Standards of the People’s Republic of China (GB/T 9695.15-2008, GB/T 5009.5-2010, and GB/T 9695.15-2008, respectively), and the results were reported on a percent (%) basis. pH value of each steak was measured directly using a portable pH meter, with the meter calibrated using two buffers (pH 4.00 and 7.00) at 4°C. The probe was inserted into each steak (about 2 cm depth) at four different locations, and pH values were averaged.

### Total viable counts

Steak surface samples (~3 mm thick slices; 10 g) were taken aseptically, chopped, and transferred to sterile lateral filter bags (BagPage; Interscience, St Nom, France), with addition of 90 mL of sterile tryptone salt solution at 0.85% (w/v). Samples were mixed with a blender (BagMixer 400; Interscience, France) for 2 min at room temperature. A 10-fold dilution series was prepared to perform microbial analysis. Diluted sample solutions were cultured on plate count agar (Land-Bridge Co., Ltd., Beijing, China) and incubated at 37°C for 48 h. Results were expressed as log colony-forming unit (CFU)/g sample.

### Color attributes

The surface color of steaks on display day 0 (blooming for 1 h at 0°C to 4°C), days 3, 5, and 7 were measured by a colorimeter (Model SP62; 8 mm diameter aperture, Illuminant A, 10° observer; X-Rite, Inc., Grand Rapids, MI, USA). The Commission Internationale de l’Eclairage L* (lightness), a* (redness), and b* (yellowness) values of each steak were measured four times and averaged. Chroma and hue values were calculated using the equation: ([a*^2^+b*^2^]^0.5^) and (arctangent [b*/a*]), respectively. The reflected wavelengths of the instrument were recorded in the range of 400 to 700 nm at 10-nm intervals and the ratio of reflectance at 630 nm and 580 nm (R630/580) was used to directly evaluate the color stability during display. The reflectance (R) at 473, 525, 572, and 700 nm were converted to reflex attenuance (A) using the equation: A = log (1/R) and the relative percentage of three myoglobin redox forms were calculated following the equations as described by AMSA [17]:

$$\begin{array}{l} \%\;\text{MMB}=\left(1.395-\frac{A572-A700}{A525-A700}\right)\times 100\\ \%\;\text{DMB}=\left[2.35\times \left(1-\frac{\text{A}473-\text{A}700}{A525-A700}\right)\right]\times 100\\ \%\;\text{OMb}=100-(\%\;\text{MMb}+\%\;\text{DMb}) \end{array}$$

### Metmyoglobin reducing activity and oxygen consumption

Several studies have reported that the initial MMb formed is a good indicator of muscle MRA [5,18]. Thus, MRA was measured as resistance to nitrite induced myoglobin oxidation to MMb. MRA determination was conducted according to the method described by Sammel et al [19] and Mancini et al [18], for which a lower initial MMb formation value indicates greater MRA. A cube (2.54×2.54×2.54 cm^3^) with no connective tissue or visible fat was removed from the central location of each steak. Then each cube was bisected horizontally, resulting in two half pieces. The top piece (including the surface exposed to light) was submerged in a 0.3% (w/v) solution of sodium nitrite for 20 min at 20°C, then was removed, blotted dry, and the reflectance of sample surface immediately measured by the colorimeter mentioned above to determine the initial MMb formation. The initial MMb formation was calculated by the formula:

$$\%\;\text{MMb}=\frac{\frac{\text{K}/\text{S}572}{\text{K}/\text{S}525}\text{ for }100\%\;\text{DMb}-\frac{\text{K}/\text{S}572}{\text{K}/\text{S}525}\text{ for sample}}{\frac{\text{K}/\text{S}572}{\text{K}/\text{S}525}\text{ for }100\%\;\text{DMb}-\frac{\text{K}/\text{S}572}{\text{K}/\text{S}525}\text{ for }100\%\;\text{MMb}}$$(AMSA [
17]).

The 100% DMb and 100% MMb were measured according to section IX B2a and section IX B1a of AMSA [17], respectively. Samples of day 0 was used to measure the 100% DMb and samples of day 3 for 100% MMb to format the most complete MMb.

Muscle OC was measured as described by Madhavi and Carpenter [20] with a modification. The freshly cut surface of the bottom half was covered with PE film as previously mentioned and bloomed for 2 h at 2°C, vacuum packaged, and immediately the reflectance of the bloomed surface was measured to determine initial OMb content by using K/S ratios and equations [17]. The packaged sample was incubated at 30°C for 30 min and then measured again to determine the final OMb content. The OC was calculated following the equation:

[(Initial OMb %-final OMb %)/initial OMb %]×100

### Lipid oxidation

Lipid oxidation was evaluated through 2-thiobarbituric acid reactive substances values according to a modified procedure of Siu and Draper [21]. Four gram of sample was homogenized for 1 min in 20 mL of distilled water by an Ultra-Turrax T18 homogenizer (T18; IKA, Staufen, Germany). Subsequently, 20 mL of 10% (w/v) trichloroacetic acid was added into the homogenate and vortex-blended, then filtered through Whatman (#1) filter paper. Amount of 1 mL of 60 mM 2-thiobarbituric acid solution was mixed with 4 mL of filtrate and incubated in a water bath at 80°C for 90 min. After the solution was cooled to room temperature, absorbance was measured using a microplate spectrophotometer (Epoch 2, Bio Tek Instruments, Winooski, VT, USA) at 532 nm and calculated with a standard curve of 1,1,3,3,-Tetraethoxypropane solution (Sigma, St. Louis, MO, USA). The results were expressed as 2-thiobarbituric acid reactive substances in mg malondialdehhyde/kg meat.

### Statistical analysis

In this experiment, statistical analysis was performed using a MIXED procedure (SAS, Version 9.0, Cary, NC, USA). Muscle type (LL and PM), ultimate pH (Np and Hp), display time and their interactions were fixed factors and carcass was a random factoProximate components for the analysis for the pH, total viable counts, color attributes, relative content of myoglobin, MRA, OC and lipid oxidation. While muscle type (LL and PM), ultimate pH (Np and Hp) and their interaction were considered as fixed factors and carcass as a random factor. Tests of differences between predicted means were applied using the PDIFF statement and differences were considered significantly different at p<0.05.

## RESULTS AND DISCUSSION

### pH, proximate composition and total viable counts

#### pH values

No interaction effect (p>0.05) of muscle type, ultimate pH (pHu) and display time was found for pH values, but there was an interaction (p<0.05) effect of pHu×muscle type on pH values (p<0.05) (Table 1), with much smaller differences within the PM muscle for the two pH groups. For both LL and PM muscles, Hp beef had a greater pH than Np beef (p<0.05), and the LL exhibited lower (p<0.05) pH values than PM from Np beef (Table 1). However, among Hp samples, LL had higher pH values (p<0.05; Table 1) than PM.

The higher pH values in the PM than those in the LL from Np beef have been reported previously [7,22]. Lefaucheur [23] reported that glycolytic muscle (such as LL) exhibited higher glycogen levels and higher actomyosin-ATPase activity compared to oxidative muscle (such as PM), which can stimulate post-mortem glycolysis and increase the accumulation of lactic acid, producing a greater decline in pH. However, more recent work has reported that oxidative muscle produces meat with a high pHu regardless of glycogen content [24]. Furthermore, PM possesses more slow-switch type I fibres, and the proportion of type I fibres is negatively correlated with pH and glycolytic indicators at 24 h postmortem [25]. Thus, the different muscle fibre composition between LL and PM of Np beef plays a decisive role in muscle pH as previously reported.

It is interesting to find that the pH of PM was lower than that of LL from Hp beef. Wulf et al [14] also reported a pH difference between LL and PM muscles of Hp (6.00 vs 5.80), but the pH of the *tensor fasciae latae and rectus femoris* from Hp beef were not different (5.66 vs 5.67). Recently, Holman and Hopkins [15] reported that the dark cutting effect on the pH of different cuts (muscles) was not uniform. However, the pH of PM was higher than LL, while the value was lower than LL from Hp beef. This inconsistency needs further exploration, possibly due to differences in glycogen content or glycogen consumption rate between muscle types or pHu groups.

#### Proximate composition

There was no interaction effect (p>0.05) between muscle type and pHu on proximate composition. While pHu had a significant effect on moisture and fat content, neither pHu nor muscle type had an effect (p>0.05) on protein content (Table 1). Hp muscle showed greater moisture (p<0.05) and lower fat content (p<0.05) than Np muscle, which is consistent with the results of English et al [12]. The higher water content in Hp beef, may be due to depletion of fat depots via increased fatty acid oxidation in Hp [26]. Also, the higher pHu of Hp muscle enhances water holding capacity, as this moves the major proteins in muscle away from the isoelectric point (pH 5.0 to 5.5), causing the proteins to have a negative charge which increases their ability to bind water. Sawyer et al [27] also documented that Hp beef showed greater bound water and lower free water levels than Np beef.

#### Total viable count

A significant pHu × muscle type × display time interaction (p<0.05) on total viable count (TVC) was found (Table 2). The initial bacterial counts for the Np-LL were higher than other samples (p<0.05). As display time extended to 7 d, TVC increased (p<0.05) significantly for all muscles. However, for both LL and PM, TVC in the Hp group were higher than the Np group on display day 7, reaching 6.61 to 6.37 log CFU/g. This finding is in agreement with previous studies, which reported that muscle with high pH was more prone to spoilage, reflecting low or absent glycogen levels of high pH muscle [10]. This is a major issue for the utilization of Hp beef.

### Color attributes

#### L* values

There was a significant pHu × muscle type interaction (p<0.05) for L* values (Table 3). L* values of Hp-LL were the lowest (p<0.05) among all samples, followed by Hp-PM, and there was no difference in L* values between Np-LL and Np-PM. In support, several previous studies have shown that Hp beef had lower L* values than Np beef, and LL from Np beef exhibited higher L* values than PM [14,27–29]. The differences in L* values between Np and Hp beef can be attributed to the pHu values. As muscle pH values increase, the water holding capacity of proteins increases, and the higher intracellular water content can cause swelling of fibers and shrinkage of the space between muscle fibrils. This decreases light scattering and increases light absorption by myoglobin. Consequently, the muscle surface color appears darker [11].

#### a*, b*, and chroma values

Significant interactions of muscle type × pHu and mscle type × display time occurred for a*, b*, chroma and hue values (Table 2). During display, all samples, except Hp-LL, showed decreased (p<0.05) a* values, and the decrease was greater in PM from both pHu beef than that in Np-LL, indicating a more stable color in LL than PM. However, a* values of Hp-LL increased (p<0.05), and the muscle remained dark, with very low L* values, as mentioned previously, which is in general agreement with previous reports [8,28,30], indicating a more stable color in LL than PM. Abril et al [31] demonstrated that a* values in Hp beef slightly increased and were greater than those in Np beef after day 6 of display, an observation not supported by the results of the current study.

The Hp-LL exhibited lower (p <0.05) a* values than Np-LL throughout display. Generally, a* values were negatively correlated (p<0.05) with muscle pH (r = −0.40; Page, 2001). McKeith et al [13] and Apple et al [32] reported that redness in LL from Hp beef was lower than that in Np beef. However, in the present study, the a* values of Hp-PM were not different from Np-PM, and the Δa* was larger in Hp samples than Np beef during display. This indicates that the color stability of Hp-PM is unaffected by the elevated pH, or maybe the pH elevation was not large enough to promote further darkening, and thus, Hp-PM is still as labile as Np-PM. Although PM from both pHu groups showed a less stable color than LL from Np beef, the a* values on day 7 were above the threshold (14.5) accepted by consumers [33].

The b* values of Np-LL remained stable (p>0.05), while b* values of PM of both normal and Hp groups decreased (p<0.05) during display. The differences in b* values of different muscles were similar to the differences in a* values. In support, Wu et al [34] reported a similar trend in both LL and PM from Np beef during storage. Joseph et al [7] reported that b* values were similar between LL and PM from Np beef during display except on day 5.

Chroma value indicates the redness intensity. In the present study, the results for chroma values for both muscles during display were similar to the surface redness. The observed chroma values in LL and PM from Np beef throughout display were in agreement with previous studies [7,30]. As for the effect of pHu, Apple et al [32] found greater chroma values in LL from Np beef than that in Hp beef, and increased chroma values in LL from Hp beef with extended display, consistent with the results reported here (Table 3).

Hue is a measure of color, with hue angle of 0 equal to red, and hue angle 90 equal to yellow (Minolta, Osaka, Japan; 1994). Both the interaction of muscle type × display time and the interaction of muscle type × pHu had effects (p<0.05) on hue (Tables 3, 4). The hue values of both LL and PM increased (p<0.05) during display, indicative of decreased redness and increased yellowness during display. Np-PM had the highest (p<0.05) hue values, followed by Np-LL and Hp-PM exhibiting similar hue values, whereas Hp-LL had the lowest (p<0.05) hue values among muscles. In agreement, McKeith et al [13] reported that as Hp severity of LL increased, the hue values decreased. Kim et al [35] documented that hue in Np-PM increased, but remained stable in LL during display. According to the results (Table 3), we confirmed that LL of both pHu groups had greater red color stability than PM, emphasizing that the effect of muscle type needs to be considered when reporting on the effect of pHu on color stability during display.

#### R630/580

R630/580 was calculated to indicate meat surface color changes during storage or retail display, in which a greater ratio means a lesser amount of MMb. As storage time increases, samples with a higher ratio have greater red color stability. There was an interaction (p<0.05) of pHu × muscle type × display time for R630/580 (Table 3). R630/580 of Np-LL was higher than other muscles and the values significantly decreased (p<0.05) in both muscles (Table 3) over time. Although PM had greater R630/580 (p<0.05) than LL on the initial day, LL muscle showed greater R630/580 (p<0.05) than PM from day 3 to 7, which was consistent with previous results [7,8]. The results for R630/580 in our study indicated a more rapid accumulation of MMb on the surface of PM steaks than on LL, reconfirming that PM is a color-labile muscle and LL is a color-stable muscle, regardless of pHu.

### Relative content of deoxymyoglobin, oxymyoglobin, and metmyoglobin

**Figure 1:**
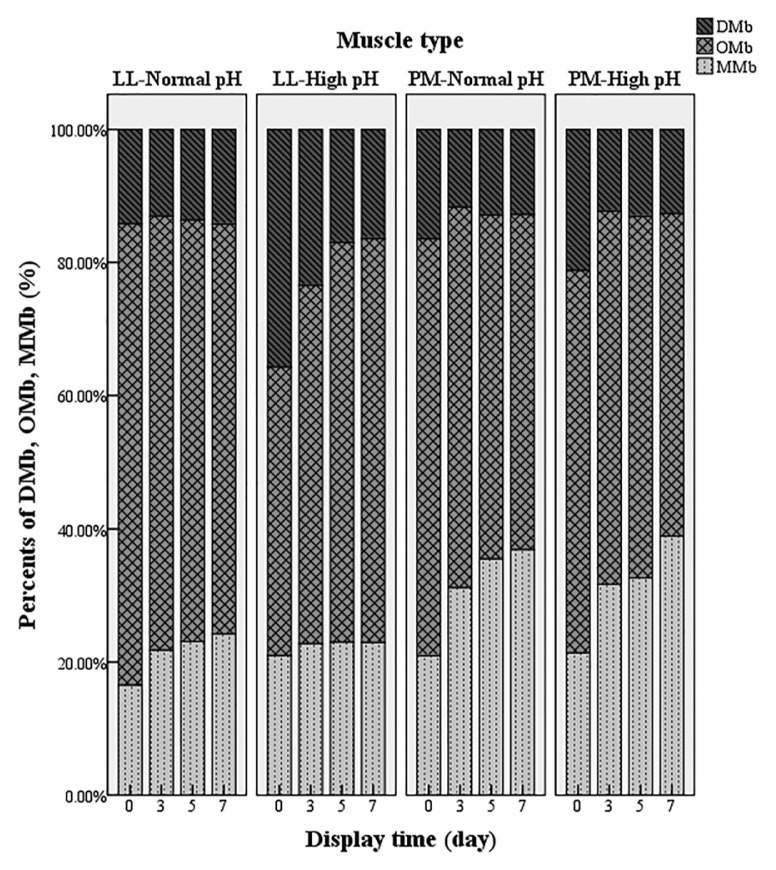
Effects of display time, muscle type (*Longissmus lumborum* [LL] vs *psoas major* [PM]) and ultimate pH (normal pH vs dark cutting) on relative content of three myoglobin forms (deoxymyoglobin, DMb; oxymyoglobin, OMb, and metmyoglobin, MMb). The content of DMb, OMb, and MMb was calculated by meat color measurement guidelines established by American Meat Science Association.

There was an interaction of pHu × muscle type × display time (p<0.05) on % DMb and % OMb. Moreover, both the interaction of muscle type × display time and the interaction of muscle type × pHu had effects on steak surface % MMb (Figure 1). During display, the % DMb of Np-LL remained stable, whereas the % DMb significantly decreased (p<0.05) from day 0 to day 3 in the other three muscles. For Hp-LL, % DMb was greatest (p<0.05) at most time points and % DMb at day 7 of display was less than half of the initial value. The results for % OMb correspond to the color values, corroborating the results of a* and chroma values. An increase (p< 0.05) of % MMb in both LL and PM was observed, and as expected, the % MMb was higher (p<0.05) in the PM than in the LL at all display days. Previous study reported that Np-PM had faster and greater % MMb accumulation and lower % OMb than Np-LL during display [5]. English et al [12] documented that Hp beef had greater % DMb than Np beef, due to high pH values which enhances mitochondrial respiration, leaving less oxygen available to bind to surface myoglobin, leading to more DMb formation [12]. The similar % OMb from PM between Np and Hp beef, resulted in the similar redness of these samples during display. However, the significantly decreased redness of Hp-PM during display was the result of the accumulation of MMb that was as high as in NP-PM.

### Metmyoglobin reducing activity

There were interactions between muscle type × display (p< 0.05) and muscle type × pHu (p<0.05) for initial metmyoglobin formation (IMF) (defined as initial MMb formation, high value indicates lower MRA; Tables 4, 5). For both LL and PM, IMF increased (p<0.05) during the first 3 days of display, with the extent more pronounced (p<0.05) in the PM, which also exhibited higher (p<0.05) value throughout display, indicating that the PM had a lower capacity to reduce MMb and poorer color stability. Furthermore, IMF and Δ% MMb were positively correlated (r = 0.96, p<0.05, data not shown). Hp-LL had lowest IMF and the IMF in the Np-PM and Hp-PM were similar (Table 5). Noticeably, IMF in Np-LL was lower than that in the Hp-PM, which is consistent with the results for R630/580 and relative MMb content, reinforcing that the color stability was greater in Np-LL than in the Hp-PM.

Previous studies in Np muscles demonstrated that MRA decreased in LL and PM muscles across display and LL exhibited greater MRA than PM, which is in agreement with our results [7,34]. Similarly, Ke et al [36] reported that LL had greater MRA than PM, and MRA in PM drastically decreased during the first three days of display. In support, English et al [12] documented that Hp-LL had greater MRA than Np-LL during ageing. The decreased MRA during display and differences between muscle types could be explained by substrate depletion, nicotinamide-adenine dinucleotide (NADH) regeneration and the activity of NADH-cytochrome b5 reductase, which is necessary for both enzymatic and non-enzymatic reducing pathways [37].

### Oxygen consumption

There was a significant interaction effect of muscle type × pHu (OC; Table 5). Compared with Hp-LL and Np-PM, Np-LL showed lower OC (p<0.05), while remarkably, the PM of Hp beef had higher (p<0.05) OC than the LL of Np beef. Previous studies have demonstrated that OC decreased in muscle with increasing days of display [5,36]. The decreased OC could be explained by the reduction in the functional ability of the mitochondria due to extended post-mortem time and depleted substrates [37]. In support, Hp beef exhibited greater mitochondrial activity and OC than Np beef, as reported previously [12]. In general, PM exhibited greater mitochondrial content and OC than LL of Np beef, as reported by Mancini et al [30]. The increased mitochondrial activity and content are responsible for the increased OC.

High OC enhances the capacity of mitochondria to com pete for available oxygen with myoglobin, resulting in more DMb or MMb formation [5,6]. It has been reported that OC plays a more crucial role in color stability, compared with MRA. Seyfert et al [6] found that if the level of MRA in the muscle could not match the oxidative stress resulting from OC, it would exhibit poorer color stability. The results presented here indicates that Np-LL, which had a similar OC to Hp-PM, showed greater color stability, all because of the much greater MRA of Np-LL than Hp-PM. But PM of both pHu groups had almost identical MRA and OC, exhibiting similar color stability.

### Lipid oxidation

The interaction of muscle type and pHu was significant for thiobarbituric acid reactive substances (TBARS) values (Table 5). Hp-LL had lower TBARS values than the other three muscles from day 3, while the significant difference was only found on day 7, and the remaining three samples did not differ (p>0.05). Hp-LL had much lower (p<0.05) fat content than Np LL (0.84% vs 1.96%, respectively; Table 1), and correspondingly lower TBARS values (0.22 vs 0.33; Table 5). The higher fat content and TBARS of the Np-LL indicates that lipid oxidation products were possibly contributing to the loss of redness observed in Np-LL at 7 days display, compared to Hp-LL.

In support of our results, Sawyer et al [27] found that LL from Np and Hp beef showed different TBARS values during the first 5-days of display, however, without a significant difference. Likewise, English et al [12] reported greater TBARS values in Np-LL than in Hp beef under vacuum packaging. Purohit et al [38] found a strong and negative correlation (−0.93) in LL between TBARS values and pH, supporting the observed results in the LL.

Similar to our findings, Jeong et al [39] documented that there was no difference in TBARS values between *Longissimus* and PM from Np beef throughout display periods, although both muscles had different fat contents. Partially in agreement with our results, several previous investigations in Np muscle reported that TBARS values increased in LL and PM over time. However, greater TBARS values were observed in PM than LL [8,36]. Generally, color-stable muscle (as LL) or/and LL muscle with high pH showed lower TBARS. Noticeably, lipid oxidation between Np-LL (color-stable muscle) and Hp-PM (high pH muscle) were not different. The reason for this is not clear.

Taken together, LL exhibited greater color stability than the PM when the pH is normal, which is attributed to the higher MMb accumulation in PM, resulting from its higher OC and lower MRA than the LL. High pH also resulted in a greater color stability, i.e. dark cutting LL had greatest color stability among all beef samples. PM from Hp beef exhibited lower color stability than LL from Np beef; this is also caused by the high accumulation of MMb, lower MRA and greater OC in the former sample. Thus, the order of color stability is LL of Hp beef, LL of Np beef, followed by PM of both pHu groups.

## CONCLUSION

Hp beef does not always show better color stability compared with Np beef, which depends on the muscle type. The balance of MRA and OC is important to keep the color in great intensity and stability in the meantime. Additionally, although redness intensity in Hp-LL was improved through display, we must consider microbiological safety and note that the TVC in Hp reached above 6.0 log CFU/g at 7-days display. These findings should aid the processing and retailing of LL and PM from different pHu beef.

## Figures and Tables

**Table 1:** Interaction effects of muscle type and ultimate pH on pH and proximate composition

Trait	LL		PM		SE
	
	Normal pH	High pH	Normal pH	High pH	
pH	5.52^d^	6.93^a^	5.77^c^	5.96^b^	0.05
Moisture (%)	73.81^b^	75.98^a^	74.45^ab^	75.27^a^	0.60
Protein (%)	22.63	22.98	23.26	22.72	0.28
Fat (%)	1.96^a^	0.84^b^	1.66^a^	1.44^a^	0.21

LL, *longissimus lumborum*; PM, *psoas major*; SE, standard error.

a–d Means in the columns with different letters differ at p<0.05.

**Table 2:** Effects of muscle type, ultimate pH and display time on total viable count

Trait	Muscle type	Ultimate pH	Display time (d)		SE

			0	7	
TVC (log CFU/g)	LL	Normal pH	2.90^a^^w^	3.88^b^^y^	0.02
		High pH	2.13^a^^x^	6.61^b^^w^	
	PM	Normal pH	2.48^a^^wx^	4.86^b^^x^	
		High pH	2.30^a^^x^	6.37^b^^w^	

SE, standard error; TVC, total viable count; CFU, colony-forming unit; LL, *longissimus lumborum*; PM, *psoas major*.

a,b Means in the rows with different letters differ at p<0.05.

w–y Means in the columns with different letters differ at p<0.05.

**Table 3:** Effects of muscle type, ultimate pH and display time on meat color

Trait	Muscle type	Ultimate pH	Display time (d)				SE

			0	3	5	7	
L*	LL	Normal pH	43.57^a^^w^	45.45^a^^w^	45.56^a^^w^	45.29^a^^w^	1.17
		High pH	32.49^a^^y^	31.88^a^^y^	32.32^a^^y^	32.42^a^^y^	
	PM	Normal pH	39.34^a^^x^	42.68^b^^w^	42.66^b^^wx^	43.79^b^^w^	
		High pH	38.26^a^^x^	38.07^a^^x^	39.72^a^^x^	38.54^a^^x^	
a*	LL	Normal pH	27.23^a^^w^	27.48^a^^w^	25.97^a^^w^	25.14^a^^w^	0.96
		High pH	17.86^a^^x^	16.01^a^^y^	17.74^a^^y^	18.07^a^^x^	
	PM	Normal pH	26.51^a^^w^	23.76^b^^x^	20.61^c^^x^	20.43^c^^x^	
		High pH	25.38^a^^w^	22.25^b^^x^	21.34^c^^x^	18.45^c^^x^	
b*	LL	Normal pH	19.16^a^^w^	20.12^a^^w^	19.12^a^^w^	18.53^a^^w^	0.87
		High pH	10.30^a^^y^	9.37^a^^y^	10.73^a^^y^	10.94^a^^y^	
	PM	Normal pH	17.70^a^^wx^	18.43^a^^wx^	16.38^a^^x^	17.01^a^^w^	
		High pH	16.53^a^^x^	16.34^a^^x^	16.05^a^^x^	14.73^a^^x^	
Chroma	LL	Normal pH	33.30^a^^w^	33.74^a^^w^	32.26^a^^w^	31.24^a^^w^	1.24
		High pH	20.66^a^^x^	18.55^a^^y^	20.74^a^^y^	21.13^a^^y^	
	PM	Normal pH	31.90^a^^w^	30.09^a^^x^	26.37^b^^x^	26.62^b^^x^	
		High pH	30.32^a^^w^	27.62^ab^^x^	26.72^bc^^x^	23.71^c^^xy^	
Hue	LL	Normal pH	35.06^a^^w^	36.18^a^^w^	36.34^a^^x^	36.38^a^^x^	0.83
		High pH	29.07^a^^y^	30.48^a^^x^	31.11^a^^y^	31.25^a^^y^	
	PM	Normal pH	33.30^a^^wx^	37.79^b^^w^	38.44^bc^^w^	39.89^c^^w^	
		High pH	32.69^a^^x^	36.18^b^^w^	36.85^b^^wx^	38.89^c^^w^	
R630/580	LL	Normal pH	4.81^a^^w^	4.31^b^^w^	3.75^c^^w^	3.75^c^^w^	0.16
		High pH	3.33^a^^x^	3.22^a^^x^	3.19^a^^x^	3.41^a^^w^	
	PM	Normal pH	5.13^a^^w^	3.33^b^^x^	2.67^c^^y^	2.59^c^^x^	
		High pH	4.83^a^^w^	3.32^b^^x^	2.84^c^^xy^	2.59^c^^x^	

SE, standard error; LL, *longissimus lumborum*; PM, *psoas major*.

a–c Means in the rows with different letters differ at p<0.05.

w–y Means in the columns within a trait with different letters differ at p<0.05.

**Table 4:** Effects of muscle type and display time on metmyoglobin reducing activity

Trait	Muscle type	Display time (d)				SE

		0	3	5	7	
IMF1)	LL	0.63^a^^x^	0.66^b^^x^	0.64^ab^^x^	0.66^ab^^x^	0.01
	PM	0.68^a^^y^	0.78^b^^y^	0.78^b^^y^	0.77^b^^y^	

MRA, metmyoglobin reducing activity; SE, standard error; IMF, initial metmyoglobin formation; LL, *longissimus lumborum*; PM, *psoas major*.

1) IMF was used to measure MRA and for which a lower value indicates greater MRA.

a,b Means in the rows with different letters differ at p<0.05.

x,y Means in the columns with different letters differ at p<0.05.

**Table 5:** Interaction effects of muscle type and ultimate pH on MRA, OC, and TBARS values

Trait	LL		PM		SE
	
	Normal pH	High pH	Normal pH	High pH	
IMF1)	0.71^c^	0.59^d^	0.74^b^	0.77^a^	0.01
OC	0.56^c^	0.64^a^	0.63^a^	0.59^b^	0.01
TBARS (malondialdehhyde mg/kg)	0.33^a^	0.22^b^	0.31^ab^	0.36^a^	0.06

MRA, metmyoglobin reducing activity; OC, oxygen consumption; TBARS, thiobarbituric acid reactive substances; LL, *longissimus lumborum*; PM, *psoas major*; SE, standard error; IMF, initial metmyoglobin formation.

1) IMF was used to measure MRA and for which a lower value indicates greater MRA.

a–d Means in the rows with different letters differ at p<0.05.
